# High‐Performance Multi‐Walled Carbon Nanotubes‐Organic Passivated Si Solar Cells Enabled by Spatially Selective Harvesting of High‐Quality Sponges

**DOI:** 10.1002/advs.76174

**Published:** 2026-07-10

**Authors:** Yuke Ren, Qi Wang, Jiahe Chen, Jianan Zhang, Jianxin Guo, Dehua Yang, Bingbing Chen, Lu Zhang, Yiming Xu, Xuan Chang, Tiansheng Sun, Qing Gao, Anyuan Cao, Jianhui Chen

**Affiliations:** ^1^ Advanced Passivation Technology Lab College of Physics Science and Technology Hebei University Baoding China; ^2^ Province‐Ministry Co‐Construction Collaborative Innovation Center of Hebei Photovoltaic Technology College of Physics Science and Technology Hebei University Baoding China; ^3^ Henan Key Laboratory of Advanced Conductor Materials Institute of Materials Henan Academy of Sciences Zhengzhou China; ^4^ State Key Laboratory of Photovoltaic Materials and Cells Yingli Group Co., Ltd Baoding China; ^5^ State Key Laboratory of Advanced Waterproof Materials School of Materials Science and Engineering Peking University Beijing China

**Keywords:** carrier‐selective transport, floating catalyst chemical vapor deposition (FCCVD), multi‐walled carbon nanotubes (MWCNTs), MWCNTs:Nafion/Si solar cells, spatial recognition of MWCNTs

## Abstract

Carbon nanotubes/Si (CNTs/Si) solar cells have emerged as promising photovoltaic devices due to their high performance and relatively simple fabrication processes. Multi‐walled CNTs (MWCNTs) in solar cells exhibit better cost‐effectiveness owing to the broader processing tolerance of MWCNTs synthesis. However, their efficiency previously reached only 12.49%, which has often been attributed to the higher defect density and inferior quality of MWCNTs compared with single‐walled CNTs. In this study, by selecting high‐quality pristine MWCNTs synthesized via floating catalyst chemical vapor deposition, we achieved a champion efficiency of 22.74% for MWCNTs:Nafion/Si solar cells. This breakthrough stems from the finding that while both the inner and outer parts of MWCNTs sponge can be uniformly dispersed when mixed with Nafion, the inner MWCNTs ink exhibits better stability than the outer MWCNTs ink due to amorphous carbon and copper impurities in the outer MWCNTs. The synergy between Nafion and the inner MWCNTs results in a high‐work‐function MWCNTs:Nafion film and a defect‐passivated Si surface, leading to favorable band alignment, excellent carrier‐selective transport properties, and consequently high device performance. This work challenges the conventional view that the higher defect density in MWCNTs inherently limits device performance and provides new insights for developing low‐cost, high‐efficiency carbon‐based/Si solar cells.

## Graphical abstract

Our work achieves an efficiency breakthrough in MWCNTs/Si solar cells by screening high‐quality pristine MWCNTs without any separation or purification processes. This breakthrough is mainly attributed to the excellent dispersibility of Nafion and the excellent hole transport property of MWCNTs:Nafion film containing charge transfer doping, passivation, and conductivity. These studies provide a feasible route for high‐performance MWCNTs/Si devices.

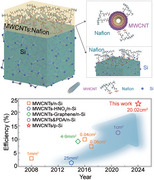

## 1 Introduction

Photovoltaic power generation has established itself as the dominant force in the renewable energy sector, serving as a core driving force in advancing global green and low‐carbon development. With breakthroughs in passivation contact technology, crystalline silicon (c‐Si) solar cells are approaching the theoretical efficiency limit of 29.4% [1] (a record efficiency of 27.81% [2]) and dominating over 95% of the global photovoltaic market. Due to the unique charge transport and optical absorption properties of graphene and carbon nanotubes (CNTs), they are frequently combined with c‐Si to form doping‐free c‐Si solar cells. Among them, CNTs exhibit high electron mobility (≈10^5^ cm^2^ V^−1^ S^−1^) [3], enormous current‐carrying capacity (>10^9^ A cm^−2^) [4], and an adjustable optical band gap, which significantly enhances the transport of charge carriers and device performance.

Based on the number of walls, CNTs are classified into single‐walled carbon nanotubes (SWCNTs), double‐walled carbon nanotubes (DWCNTs), and multi‐walled carbon nanotubes (MWCNTs). The synthesis of SWCNTs and DWCNTs requires more critical control over the size and crystallographic orientation of catalyst nanoparticles (e.g., Fe, Co [5, 6]) and the process of separation and purification, which enables better charge carrier transport properties and a wide range of the solar spectrum of SWCNTs and DWCNTs. These properties make them a perfect hole‐transport material, albeit at the expense of lower production yield. In 2007 [7], Tsinghua University first reported DWCNTs/Si heterojunction solar cells, which set a precedent for CNTs/Si solar cells research. To further improve the properties of CNTs/Si solar cells, strategies for doping (HNO_3_, poly (methyl methacrylate) (PMMA) [8]), interfacial passivation, and anti‐reflection layer (PDMS [9], MgF_2_) are studied. Due to the excellent electrical and optical properties of SWCNTs, the SWCNTs/Si photovoltaic device has obtained a high fill factor (FF) of 73.8% and an ideality factor of 1.08 [10]. MoO_x_ and ZnO were used to reduce the Schottky barrier of SWCNTs/Si interface and doped CNTs, resulting in a high efficiency of 17% and 4%, respectively [11]. However, fluid processing of CNT film makes it easily crumpled and folded, which limits the area of CNTs/Si solar cells (typically <0.15 cm^2^). Cao et al. increased the active area to 2 cm^2^ by adding highly conductive CNT strips to the surface of solar cells, and achieved an efficiency of about 10% [12]. In 2016, Chen et al. found the passivation function of the sulfonic acid group. After Nafion and PSS passivated the Si surface defects, the minority carrier lifetime (*τ*_eff_) of 9.6 ms [13] and 28.6 ms [14] were obtained, respectively, reaching the passivation level of SiO_2_ and a‐Si:H films with the best passivation effect in the current photovoltaic field. Therefore, one layer of CNTs: organic composite film has the dual function of conduction and passivation, which makes it possible to achieve large area preparation of CNTs/Si solar cells. In 2020, efficiencies of 21.4% on an SWCNTs/Si device area of 4.8 cm^2^ and 20% on an industrial size (245.71 cm^2^) wafer were obtained [15]. SG65i SWCNTs/Si showed a good contact and a low reverse saturation current density (*J*_0_) with a highest efficiency of over 23% [16]. However, the stringent fabrication conditions and complicated purification processes of SWCNTs or DWCNTs pose significant challenges for the low‐cost and large‐scale production of CNTs/Si solar cells.

Compared with SWCNTs and DWCNTs, MWCNTs can be mass‐produced under less‐stringent and inexpensive synthesis conditions without requiring complex chiral separation and purification processes [17, 18]. The band gap of MWCNTs narrows or disappears as the number of walls increases, usually exhibiting metallic properties. The interwall coupling effects in MWCNTs endow them with excellent physical properties, including ballistic transport [19, 20, 21], negative magnetoresistance [22, 23], and quantum interference effects [24, 25, 26]. In general, MWCNTs' interwall coupling interactions in MWCNTs are relatively weak due to van der Waals forces between layers [27]. However, strategic modulation of material characteristics (e.g., crystallinity, defect density, diameter control, and external magnetic fields) can effectively enhance interlayer coupling intensity, modifying the optoelectronic properties of MWCNTs. In addition, functionalization of the MWCNTs' outer walls can modify their surface electronic structure, thereby influencing their optoelectronic properties. Therefore, MWCNTs exhibit significant potential as inexpensive yet high‐performance photovoltaic materials. However, the performance of MWCNTs/Si solar cells is relatively low (Table S1). In 2014, Chen et al. [28] reported a MWCNTs/Si heterojunction solar cell, demonstrating that acid functionalization of MWCNTs outer walls forms a Schottky‐like heterojunction with silicon, achieving an efficiency of 1.39%. In 2015, Cao et al. [29] found that a graphene‐MWCNTs hybrid network could enhance thin film conductivity, achieving a power conversion efficiency (PCE) of 9.24%. In 2016, Nicola et al. [30] fabricated MWCNTs/Si solar cells using a dry transfer printing process, achieving a 10% PCE. Their study revealed that introducing an interfacial silicon oxide layer on the Si surface enhances device stability (maintaining performance without degradation after 30 days in ambient air). In 2018, Cao et al. [31] demonstrated that embedding metal nanoparticles (e.g., Au, Pt) into MWCNT networks enhances the light absorption and reduces bulk resistivity, achieving a PCE of 7.4%. In 2021, MWCNTs coated with polydopamine were applied as an interlayer to enhance the performance of Si/PEDOT: PSS junctions, achieving a champion PCE of 12.49% [32]. It can be seen that the performance of MWCNTs/Si solar cells is much lower than that of SWCNTs/Si solar cells. The conventional perception is that MWCNTs have more defects and poorer quality than that of SWCNTs.

This work obtains a champion efficiency for the MWCNTs: Nafion/Si solar cells and conducts a detailed analysis of the factors influencing the performance of MWCNTs: Nafion/Si solar cells. First, the spatial recognition is conducive to obtaining high‐quality MWCNTs easily. The interior and exterior parts of the MWCNTs sponge, by floating catalyst chemical vapor deposition (FCCVD), can form a uniformly dispersed ink when mixed with Nafion. However, the interior MWCNTs ink has better stability than the exterior MWCNTs ink due to the presence of amorphous carbon and Cu impurities in the exterior MWCNTs. In addition, the synergistic effect of Nafion and MWCNTs results in a high work function of 5.52 eV for the MWCNTs: Nafion film, which provides a proper band alignment with Si. Combined with the excellent passivation effect of Nafion, these results demonstrate that the MWCNTs: Nafion film has excellent carrier‐selective transport properties. Moreover, the mechanisms of MWCNTs nucleation, growth kinetics, defect formation, and electronic structure are analyzed in detail to understand their impact on the device performance and characteristics of MWCNTs, including morphology, I_G_/I_D_ ratio, conductivity, and nanotube diameter under different growth conditions. These studies provide a feasible route using MWCNTs to construct high‐performance MWCNTs: Nafion/Si solar cells.

## 2 Results and Discussion

**FIGURE 1:**
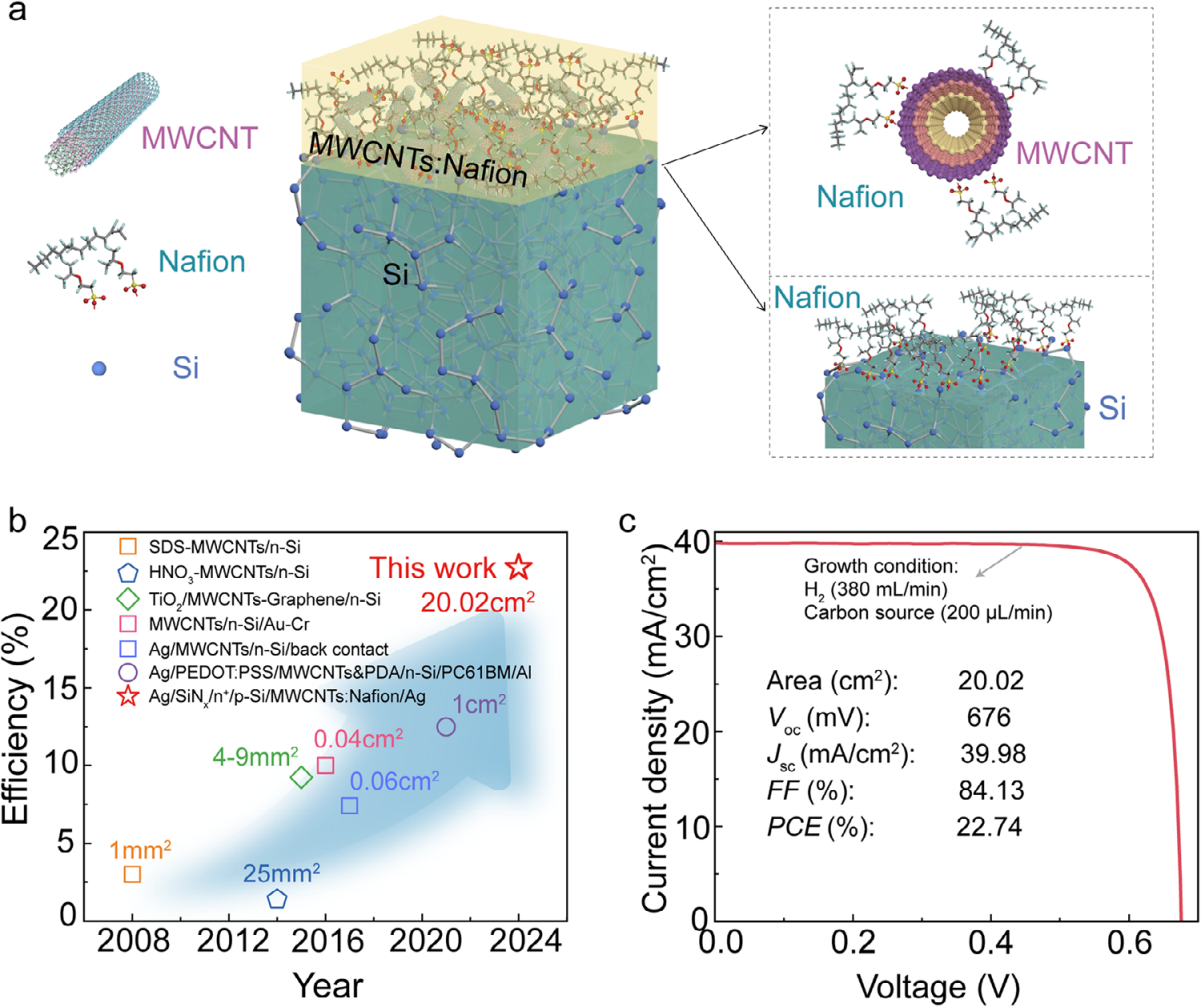
a) Schematic diagram of the function of the MWCNTs: Nafion film on Si. b) Historical progression of MWCNTs/Si solar cells over the past few decades. c) Current‐voltage (*J‐V*) curve and optimal photovoltaic performance parameters of the MWCNTs: Nafion/Si solar cells.

MWCNTs (Figure 1a) prepared by FCCVD are mixed with the organic solvent Nafion to form an MWCNTs: Nafion film. The MWCNTs: Nafion film has excellent hole‐selective transport properties, containing a high work function and the interface defect passivation, which is attributed to the synergistic effect of Nafion and MWCNTs. Nafion demonstrates multi‐functional properties: 1) dispersing MWCNTs; 2) passivating the defects of MWCNTs to increase the work function of the MWCNTs, Nafion film by the synergistic effect of Nafion and MWCNTs; 3) passivating the Si surface defects. The MWCNTs: Nafion film coated with Ag electrode is used as the back surface field of MWCNTs: Nafion/Si solar cells (device architecture in Figure S1). The front structure of MWCNTs: Nafion/Si solar cells is stacked by Ag/SiN_x_/n^+^ emitter layers. The MWCNTs: Nafion/Si solar cells use a p‐type Czochralski‐grown (Cz) boron‐doped silicon wafer with a resistivity of about 2 Ω·cm to produce photogenerated charge carriers. The highest PCE of 22.74% (Figure 1b,c; Figure S2) is achieved in the MWCNTs: Nafion/Si solar cells, which is significantly higher than the current maximum PCE of 12.49% [32] for the MWCNTs/Si solar cells. The PCE of MWCNTs: Nafion/Si solar cells shows good stability after about 200 h at atmospheric environment. Especially *V*_OC_ and FF are maintained above 99% of the initial values (Figure S3). When MWCNTs: Nafion film is used as the transparent hole transport layer on the upper layer of the MWCNTs: Nafion/Si solar cells, the corresponding device performance is a PCE of 17.76% with a *V*_OC_ of 619 mV, a *J*_SC_ of 39.69 mA cm^−2^, and a FF of 72.30% (Figure S4). It is worth noting that MWCNTs did not undergo any separation or purification processes. These data demonstrate that the MWCNTs: Nafion/Si solar cells possess the advantage of a simple fabrication process and the potential to achieve high efficiency.

**FIGURE 2:**
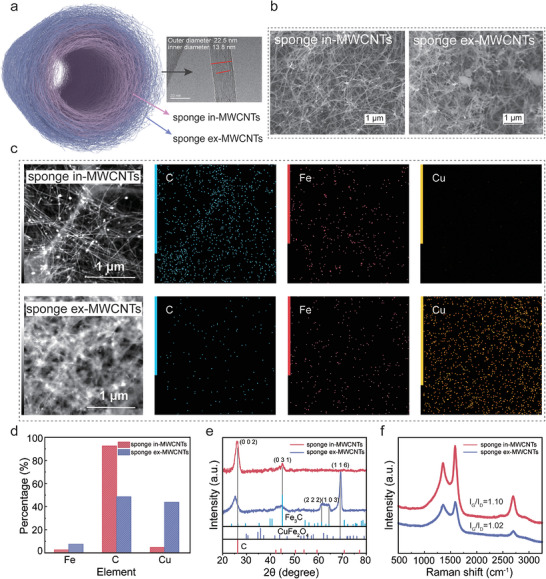
a) Schematic diagram of the MWCNTs sponge structure and TEM image of a single MWCNT. b) SEM images of in‐MWCNTs and ex‐MWCNTs. c, d) TEM images of the in‐MWCNTs and ex‐MWCNTs and corresponding EDS images of C, Fe, and Cu elements. e) XRD spectra of the in‐MWCNTs and ex‐MWCNTs. f) Raman spectra of the in‐MWCNTs and ex‐MWCNTs.

Transmission electron microscope (TEM) measurement (Figure 2a) shows that the outer diameter of MWCNTs by FCCVD is approximately 22.5 nm, and the inner diameter is 13.8 nm. Based on the CNT layer spacing of 0.34 nm, the number of tube walls of MWCNTs is about 14. The MWCNTs prepared by FCCVD exhibit a tubular shape and sponge‐like structure (as illustrated in Figure 2a). In order to study the influence mechanism of MWCNTs: Nafion/Si solar cells, the properties of the MWCNT sponge are first investigated. The interior of the MWCNTs sponge is defined as the in‐MWCNTs (purple MWCNTs in Figure 2a), and the exterior is defined as the ex‐MWCNTs (blue MWCNTs in Figure 2a). The scanning electron microscope (SEM) measurements of these two parts of MWCNTs (Figure 2b) show that the in‐MWCNTs are clearly distinct one by one, indicating that their impurity content is relatively low. However, the ex‐MWCNTs contain many more impurities with irregular particle shapes. Energy dispersive X‐ray spectroscopy (EDS) element mapping of TEM measurements (Figure 2c,d) shows that the C contents of the in‐MWCNTs and ex‐MWCNTs are 92.62% and 48.58%, respectively; Fe contents are 2.58% and 7.51%, respectively; and Cu contents are 4.8% and 43.91%, respectively. These results indicate that the ex‐MWCNTs contain more Cu and Fe impurities. X‐ray Diffraction (XRD) measurements (Figure 2e) measurements of MWCNTs show that the main peaks of in‐MWCNTs and ex‐MWCNTs are C phase (at 26.2°), CuFe_2_O_4_ phase (at 69.1°), respectively. These results indicate the presence of Cu impurities, especially in ex‐MWCNTs, which is consistent with the results of SEM measurements. Raman spectra of the in‐MWCNTs and ex‐MWCNTs display two main bands at 1330 cm^−1^ (D band) and 1597 cm^−1^ (G band). D band represents a disordered MWCNT lattice contributed from the edge of the MWCNT or sp^3^ hybridized (C─C). The G band represents the sp^2^ hybridized of an ideal MWCNT vibration mode (C = C). The intensive ratio of I_G_/I_D_ for the in‐MWCNTs and ex‐MWCNTs is 1.10 and 1.02 (Figure 2f), respectively. The values indicate that the ex‐MWCNTs have more defects and probably a higher amount of amorphous carbon, which will affect the electronic distribution and work function of MWCNTs.

**FIGURE 3:**
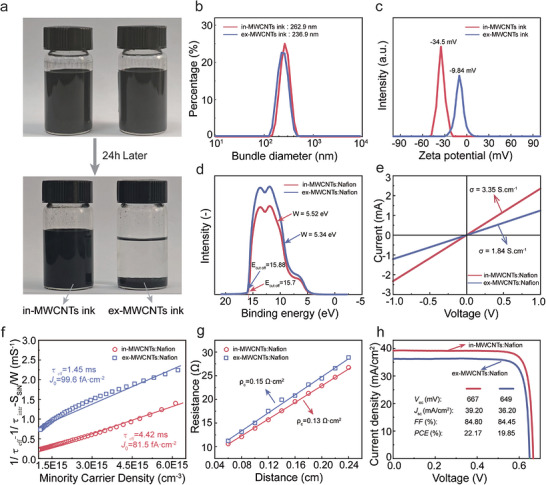
a) In‐MWCNTs and ex‐MWCNTs inks before and after 24 h. b‐c) Particle size distribution and Zeta potential of the in‐MWCNTs and ex‐MWCNTs inks. d) *W*_F_ of in‐MWCNTs: Nafion and ex‐MWCNTs: Nafion films. e) Conductivity test using a coplanar electrode configuration. f) *τ*_eff_ (the evolution of lifetime at a minority carrier density of 10^15^ cm^−3^) of the high resistivity silicon wafer coated with in‐MWCNTs: Nafion and ex‐MWCNTs: Nafion film. g) Contact resistance values (*ρ*_c_) of in‐MWCNTs: Nafion/Si and ex‐MWCNTs: Nafion/Si measured by transmission line model (TLM) method. h) *J‐V* curves of in‐MWCNTs: Nafion/Si and ex‐MWCNTs: Nafion/Si solar cells and the optimal photovoltaic performance parameters.

From MWCNTs sponge to MWCNTs: Nafion/Si solar cells, the first step is to disperse the MWCNTs in a solvent. The in‐MWCNTs and ex‐MWCNTs are respectively mixed with 5.88 wt.% Nafion solution to prepare two types of MWCNTs ink by a high‐pressure blasting system in Figure 3a. The dispersion of the MWCNTs (Figure 3b) by dynamic light scattering (DLS) shows two narrow peaks. The average bundle size of the in‐MWCNTs and ex‐MWCNTs is 263 and 237 nm, respectively. These results indicate that both of these MWCNTs exhibit good dispersion, and the average bundle size of the ex‐MWCNTs ink is smaller than that of the in‐MWCNTs ink, which is because the ex‐MWCNTs contain more impurities and defects. Zeta potential measurements are carried out to analyze the stability of the dispersion system (Figure 3c). The Zeta potential of the in‐MWCNTs and ex‐MWCNTs is −34.5 and −9.84 mV, respectively, certifying that the in‐MWCNTs ink is more stable than that of the ex‐MWCNTs. After leaving the two dispersed solutions in Figure 3a for 24 h, it was observed that the in‐MWCNTs ink remained unchanged, while the ex‐MWCNTs ink showed significant stratification. The ex‐MWCNTs completely settle at the bottom of the ink, which is attributed to more impurities of the ex‐MWCNTs.

Since MWCNTs are naturally p‐type materials, they can be utilized to extract and transport holes in Si solar cells. Therefore, the in‐MWCNTs: Nafion and ex‐MWCNTs: Nafion films prepared from the in‐MWCNTs and ex‐MWCNTs inks are required to possess three outstanding properties: 1) high work function (*W*_F_), which forms the transmission barrier for electrons and the transmission channel for holes; 2) passivation effect, which can passivate the surface defects of Si surface and reduce the loss of interface recombination; 3) high electrical conductivity, which enables the efficient transmission of holes to the electrode. Figure 3d shows that *W*_F_ of the in‐MWCNTs and ex‐MWCNTs: Nafion films are 5.52 eV and 5.34 eV, respectively. In general, the *W*_F_ of the MWCNT sponge is 4.39 eV (Figure S5). The higher *W*_F_ of the in‐MWCNTs and ex‐MWCNTs: Nafion films is mainly due to the synergistic effect of Nafion on MWCNTs. First‐principles calculations (Figure S6) show that the H of Nafion can bond with the C of MWCNTs, accompanied by obvious electron exchange. Meanwhile, the H atom from Nafion can passivate the defects of MWCNTs. This interaction reveals the increased hole concentration and *W*_F_ of MWCNTs: Nafion films [33]. The difference in the *W*_F_ between the in‐MWCNTs and ex‐MWCNTs: Nafion films is mainly due to the different defect density and impurity content. The *W*_F_ of the in‐MWCNTs: Nafion films is higher than that of the ex‐MWCNTs: Nafion films. The higher *W*_F_ is more conducive to the selective transport of holes and the improvement of device performance. The conductivity of the in‐MWCNTs: Nafion is larger than that of ex‐MWCNTs: Nafion film (Figure 3e), which is because the higher defect density of ex‐MWCNTs has deteriorated the conductivity. This indicates that in‐MWCNTs: Nafion film has a better hole transport capability.

The minority carrier lifetime (*τ*_eff_) measured by transient photoconductance decay (PCD) on high resistivity Si wafers shows that a millisecond‐level *τ*_eff_ is obtained for both in‐MWCNTs: Nafion and ex‐MWCNTs: Nafion films, indicating that the oxygen in the sulfonic function group (‐SO_3_^−^) of Nafion can effectively passivate defects on the Si surface by an electrochemical passivation mechanism [34]. In particular, *τ*_eff_ of the in‐MWCNTs: Nafion film is 4.42 ms, which is higher than that of 1.45 ms of the ex‐MWCNTs: Nafion film (Figure 3f). The result is mainly attributed to the metal impurities of the ex‐MWCNTs, which, to some extent, affected the passivation effect of the Nafion sulfonic function group. Similarly, the low content of metal impurities of the in‐MWCNTs also results in a lower contact resistance and saturation current density (*J*_0_) (Figure 3g) between in‐MWCNTs: Nafion and Si than those between ex‐MWCNTs: Nafion and Si. In summary, the MWCNTs: Nafion films prepared by dispersing MWCNTs in the organic solvent Nafion not only have a high *W*_F_, but also can effectively passivate the Si surface defects to form a good contact. The corresponding performances of in‐MWCNTs: Nafion/Si and ex‐MWCNTs: Nafion/Si solar cells (Figure 3h) are the power conversion efficiency (PCE) of 22.17% and 19.85%, the open‐circuit voltage (*V*_OC_) of 667 and 649 mV, the fill factor (FF) of 84.80% and 84.45%, the short‐circuit current density (*J*_SC_) of 39.2 and 36.2 mA cm^−2^, respectively. The device performances show that in‐MWCNTs: Nafion/Si solar cells have a higher *V*_OC_, *J*_SC_, and PCE than those in the ex‐MWCNTs: Nafion/Si solar cells. To understand the role of MWCNTs, a reference experiment without MWCNTs: Nafion film shows that the PCE of the reference solar cells without MWCNTs: Nafion film is only 16.66% with a *V*_OC_ of 620 mV, a *J*_SC_ of 38.42 mA cm^−2^, and a FF of 69.95% (Figure S7), which is much lower than that of the MWCNTs: Nafion/Si solar cells. Another reference experiment with Nafion film replacing the MWCNTs: Nafion film shows 16.98% with a *V*_OC_ of 612 mV, a *J*_SC_ of 35.90 mA cm^−2^, and a FF of 77.25%. These results contribute to a high‐quality defect passivation and excellent hole extraction and transport from high work function in the in‐MWCNTs: Nafion/Si solar cells.

**FIGURE 4:**
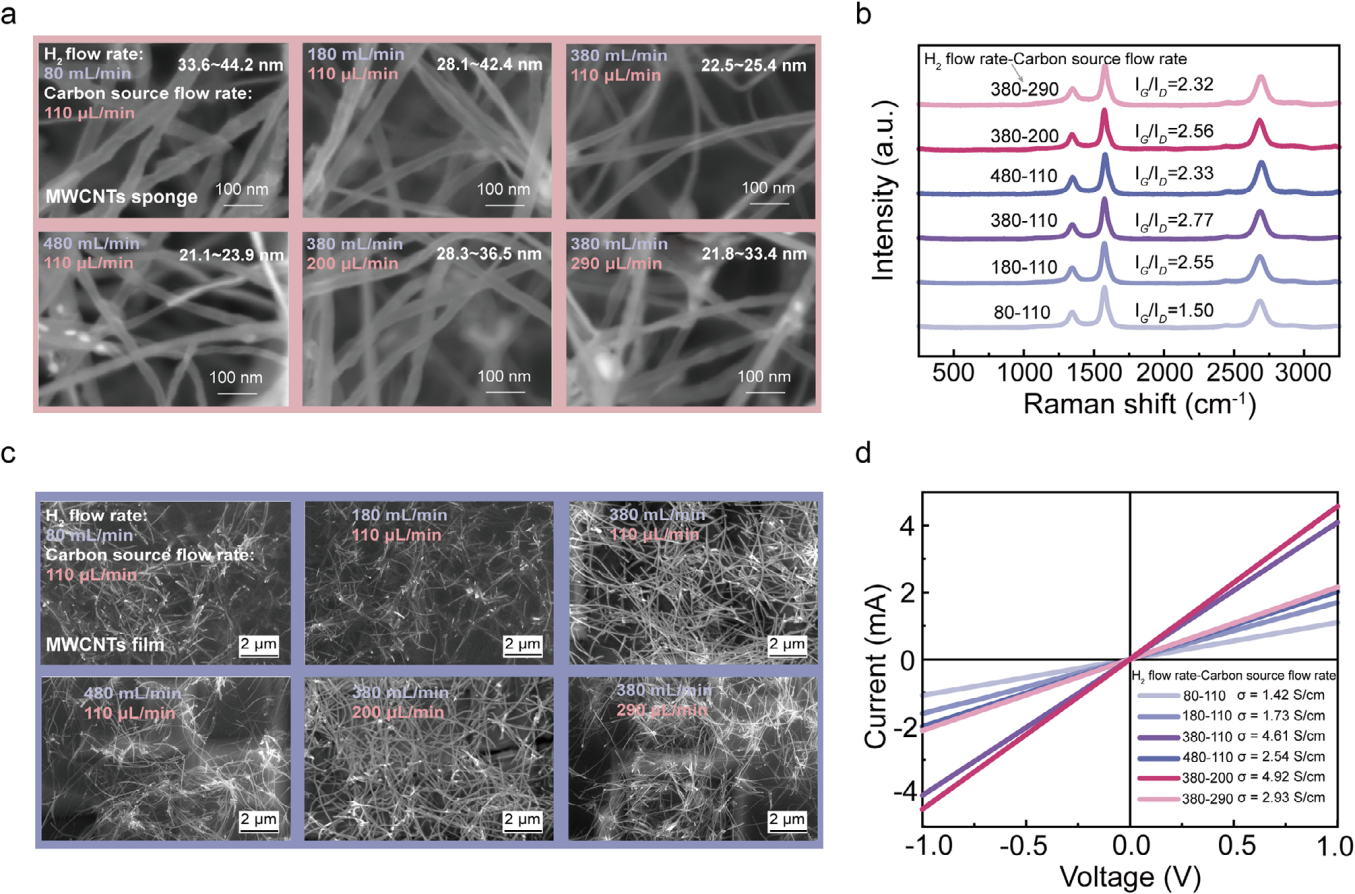
a) SEM images and b) Raman spectra of MWCNTs sponges prepared by different H_2_ and carbon source flow rates. c) SEM images and d) conductivity measurement using Ag coplanar electrode configuration of the corresponding MWCNTs: Nafion films.

Furthermore, to achieve high‐performance MWCNTs: Nafion/Si solar cells with excellent reproducibility and industrial feasibility, it is necessary to conduct a detailed analysis of various factors affecting the performance of MWCNTs/Si devices, such as nanotube diameter, I_G_/I_D_ ratio, and defects. These factors are influenced by the preparation conditions of MWCNTs. The main preparation conditions of MWCNTs contain the H_2_ flow rate and the carbon source flow rate, whose effects on the MWCNTs structure are closely related to the underlying growth mechanism. The formation of the MWCNTs sponge follows an “open‐ended growth+initial gas‐phase growth” model within a typical vapor–liquid–solid (VLS) mechanism [35, 36]. In brief, ferrocene first decomposes in the gas phase to generate Fe atoms, which nucleate and grow into liquid Fe nanoparticles. Subsequently, the carbon precursor is adsorbed and decomposed on the catalyst surface, followed by carbon diffusion within the catalyst and precipitation as MWCNTs upon supersaturation (Figure S8). Therefore, the effects of the H_2_ flow rate and carbon source flow rate on MWCNTs structure can be fundamentally understood as their coupled regulation of this rate‐limiting adsorption/decomposition process of carbon species on the catalyst surface. In the FCCVD process, these parameters modulate the local concentrations of catalytically active Fe species and carbon‐containing species in the reaction zone, ultimately determining the nanotube structure. These structural variations further affect the electronic structure and charge transport behavior of the MWCNTs network through changes in π‐electron delocalization, defect‐induced carrier scattering, and intertube contact resistance, and thus govern the conductivity of the MWCNTs: Nafion film and the final device performance. SEM images of MWCNTs with a variable carbon source flow rate and a variable H_2_ flow rate reflect the changes in the structure and properties of MWCNTs (Figure 4a). When the H_2_ flow rate is 80 mL min^−1^ and the carbon source flow rate is 110 µL min^−1^, MWCNTs with a diameter of 33.6–44.2 nm and I_G_/I_D_ ratio of 1.50 (Figure 4b) are obtained. Similar bamboo‐like structures are observed, indicating more defects on the walls of MWCNTs. These defects will affect the continuity of MWCNTs, which is confirmed in the corresponding MWCNTs: Nafion film (Figure 4c). The MWCNTs resembling short branches are arranged in a crisscross and stacked manner in the MWCNTs: Nafion film. It is well known that the inter‐tube resistance of CNTs is a key factor affecting the conductivity [37]. Therefore, the conductivity of the MWCNTs: Nafion film with 80 mL min^−1^ of H_2_ flow rate and 110 µL min^−1^ of carbon source flow rate is low (1.42 S cm^−1^) in Figure 4d, which will affect the hole‐transport and the device performance. At this relatively low H_2_ flow rate, the concentration of active carbon species in the reaction zone is high, which tends to cause rapid local carbon deposition and disordered growth. As a result, bamboo‐like structures and high defect density are more likely to form, leading to poor nanotube continuity and inferior film conductivity. When the H_2_ flow rate was increased to 380 mL min^−1^ while keeping the carbon source flow rate (110 µL min^−1^) constant (Figure 4a), the bamboo‐like structure of MWCNTs gradually decreased, accompanied by an average diameter of 22.5–25.4 nm and I_G_/I_D_ ratio of 2.77 (Figure 4b). This indicates a reduction in defects and an enhancement in continuity of the MWCNTs. The corresponding MWCNTs: Nafion film (Figure 4c) shows a continuing network of MWCNTs, which provides a continuous channel for the hole‐transport and reduces the influence of the inter‐tube resistance of CNTs on the conductivity. The corresponding conductivity of the MWCNTs: Nafion film increases to 4.61 S cm^−1^. This indicates that an appropriate H_2_ flow can suppress disordered carbon deposition and help maintain catalyst activity, thereby favoring ordered and continuous CNTs growth. Under this condition, the balance among catalyst stability, carbon supply, and axial nanotube growth is optimized. Continuing to increase the H_2_ flow rate to 480 mL min^−1^, the bamboo‐like structure reappeared, and I_G_/I_D_ ratio reduced to 2.33 (Figure 4b). Simultaneously, the continuity of the corresponding MWCNTs decreased (Figure 4a), and the conductivity of the MWCNTs: Nafion film also decreased to 2.54 S cm^−1^ (Figure 4d). At excessively high H_2_ flow, more Fe species are rapidly removed from the effective reaction zone, lowering the local Fe concentration and suppressing catalyst coalescence into larger particles. Meanwhile, overly high H_2_ also weakens effective precursor decomposition and carbon supply, limiting sustained axial growth and inducing the reappearance of bamboo‐like structures. These data reflect that an H_2_ flow rate of 380 mL min^−1^ is optimal for synthesizing MWCNTs with fewer defects and a continuous structure. Therefore, the H_2_ flow rate of 380 mL min^−1^ was kept constant, while the influence of carbon source flow rate was systematically investigated changed. When the carbon source flow rate increased to 200 µL min^−1^, the MWCNTs exhibited a conductivity of 4.92 S cm^−1^ and an I_G_/I_D_ ratio of 2.56, and an increased diameter in the range of 28.3–36.5 nm. Increasing the carbon source flow rate raises the concentrations of both Fe species and carbon‐containing species in the reaction zone. The higher Fe concentration facilitates catalyst collision, coalescence, and growth into larger particles, while the enhanced carbon supply promotes the continuous precipitation of additional graphitic walls. Therefore, MWCNTs with larger diameters and thicker walls are obtained. Continue to increase the carbon source flow rate to 290 µL min^−1^, the bamboo‐like structure becomes more pronounced, and the average nanotube diameter and the I_G_/I_D_ ratio decrease to 21.8–33.4 nm and 2.32, respectively. Meanwhile, the continuity of the corresponding MWCNTs decreased (Figure 4a), and the conductivity of the MWCNTs: Nafion film also decreased to 2.93 S cm^−1^ (Figure 4d). When the carbon source flow rate is excessively high, the carbon flux to the catalyst surface becomes too large, which promotes radial deposition and wall accumulation and gradually drives the system away from the stable growth regime. It leads to rougher outer walls, more pronounced bamboo‐like morphology, deteriorated nanotube continuity, and reduced film conductivity. Therefore, the existence of optimal H_2_ and carbon source flow rates is not merely an empirical observation, but reflects the competition among catalyst size evolution, surface adsorption/decomposition kinetics, and carbon precipitation balance during MWCNTs growth. The cross‐sectional SEM image of the MWCNTs: Nafion film (Figure S9) shows a relatively uniform film and the thickness of about 200 nm.

**FIGURE 5:**
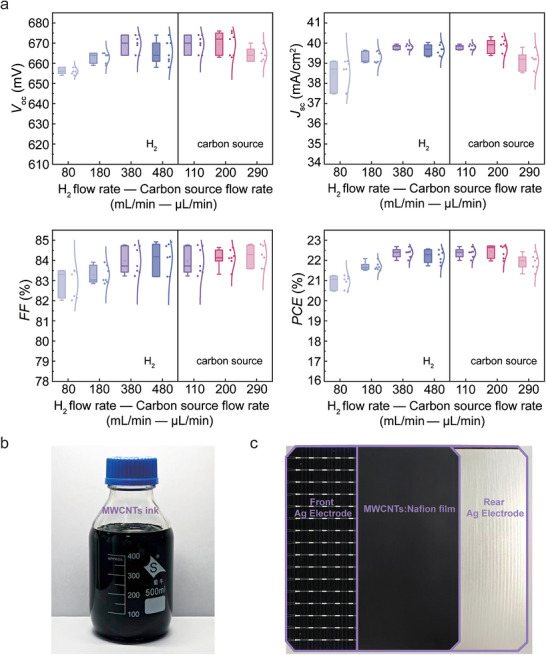
a) Device performances of MWCNTs: Nafion/Si solar cells based on MWCNTs grown under different H_2_ flow rate and carbon source flow rate. Each condition contains 7 solar cells. b) MWCNTs ink. c) The front surface and the rear MWCNTs: Nafion film and Ag electrode morphology of the device.

The same device structure as mentioned above is applied to study the performances of MWCNTs: Nafion/Si solar cells (Figure 5a, Table S2). When the carbon source flow rate is 110 µL min^−1^, the *V*_OC_ and the *J*_SC_ increase as the H_2_ flow rate increases. While the FF increased with H_2_ flow rate up to 380 mL min^−1^, no further improvement is observed at higher flow rates. A high PCE of 22.68% with a *V*_OC_ of 672 mV, a *J*_SC_ of 39.85 mA cm^−2^, and a FF of 84.72% is achieved at a H_2_ flow rate of 380 mL min^−1^. When H_2_ flow rate is 380 mL min^−1^, the *V*_OC_ and the *J*_SC_ first increase and then decrease as the carbon source flow rate increases. Finally, the champion PCE of 22.74% with a *V*_OC_ of 676 mV, a *J*_SC_ of 39.98 mA cm^−2^, and a FF of 84.13% is achieved at H_2_ flow rate of 380 mL min^−1^ and a carbon source flow rate of 200 µL min^−1^. In addition, it is worth noting that the PCE of all the MWCNTs: Nafion/Si solar cells exceeds 20%, which is much higher than the highest PCE reported for MWCNTs/Si solar cells to date, and also demonstrates the potential of MWCNTs: Nafion /Si solar cells. MWCNTs with a more relaxed preparation process window enable facile large‐scale production of kilogram‐scale inks (Figure 5b) and industrial‐sized MWCNTs: Nafion/Si solar cells (Figure 5c). The acquisition of high‐performance and highly reproducible MWCNTs: Nafion/Si solar cells challenges the notion that MWCNTs' defects limit device performance, paving the way for low‐cost, high‐efficiency carbon/Si solar cells.

## 3 Conclusion

In this work, record‐efficiency MWCNTs: Nafion/Si solar cells are obtained from MWCNTs without any separation or purification process. First, the spatial recognition of high‐quality MWCNTs prepared by the FCCVD method is thoroughly studied. The Cu impurities in ex‐MWCNTs have an impact on the stability of MWCNTs ink and the work function of the MWCNTs: Nafion film. The in‐MWCNTs ink demonstrates better stability than the ex‐MWCNTs ink after 24 h. The synergistic effect of Nafion and MWCNTs results in a higher work function of 5.52 eV of the in‐MWCNTs: Nafion film than that (5.34 eV) of the ex‐MWCNTs: Nafion film. Nafion among the MWCNTs: Nafion/Si solar cells have another function: dispersing MWCNTs. These properties result in that in‐ MWCNTs: Nafion/Si solar cells exhibit better interface passivation (*τ*_eff_ = 4.42 ms) and hole‐selective transport properties than those of ex‐ MWCNTs: Nafion/Si solar cells. In addition, a detailed analysis is conducted on the factors influencing the performance of MWCNTs: Nafion/Si solar cells, including MWCNTs morphology, I_G_/I_D_ ratio, conductivity, and nanotube diameter from the growth conditions of MWCNTs. When the I_G_/I_D_ ratio of MWCNTs is higher, MWCNTs exhibit better continuity and higher conductivity, resulting in a higher performance of MWCNTs: Nafion/Si solar cells. While the diameter of MWCNTs has no direct relationship with the performance of MWCNTs/Si devices. It is worth noting that the PCE of all the MWCNTs: Nafion/Si solar cells exceeds 20%, which is much higher than the highest PCE reported for MWCNTs/Si solar cells to date. Finally, the champion PCE of 22.74% with a *V*_OC_ of 676 mV, a *J*_SC_ of 39.98 mA cm^−2^, and a FF of 84.13% was achieved. These results demonstrate the potential of MWCNTs: Nafion/Si solar cells by inexpensive nanomaterials and simplified processes. This work offers new ideas for low‐cost and high‐efficiency carbon/Si solar cells.

## 4 Experimental Section

### 4.1 Preparation of MWCNTs Sponge

The MWCNT sponge was synthesized via FCCVD. An integrated precursor solution was prepared by dissolving 0.05 g mL^−1^ ferrocene (Shanghai D&B, K331039) in 1,2‐dichlorobenzene (Aladdin, D108140), where in ferrocene served as the catalyst precursor and 1,2‐dichlorobenzene acted as the carbon source. The precursor was injected into a quartz tube using a syringe pump under a reducing atmosphere of 15% H_2_/Ar at 840 °C. After a reaction time of 30 min to 4 h, a free‐standing monolithic MWCNTs sponge was obtained on the Cu substrate placed in the central region of the quartz tube.

### 4.2 Preparation of the MWCNTs/Si Solar Cells

A 5.88 wt.% Nafion solution was prepared by mixing the 20 wt.% Nafion (Sigma–Aldrich, 20 wt.% in a mixture of lower aliphatic alcohols and 34% water) with ethanol (Sigma–Aldrich, 20 proof anhydrous, ≥99.5%). A uniform precursor solution was obtained through magnetic stirring for at least 4 h. The in‐MWCNTs (purple MWCNTs in Figure 2a) and the ex‐MWCNTs (blue MWCNTs in Figure 2a) were separated by a physical method (using forceps). Then, different MWCNTs were mixed in the Nafion solution at a ratio of 2 mg mL^−1^, and the solution performed magnetic stirring for at least 3 h. A uniform MWCNT solution needed to undergo secondary dispersion through a high‐pressure blasting system, which utilizes the pore effect and shear effect generated by the high‐pressure difference to achieve good dispersion of the MWCNTs ink. This dispersion method of the solution has a high repeatability. Depending on the type of MWCNTs, uniform dispersed in‐MWCNTs and ex‐MWCNTs solutions can be prepared. A boron‐doped p‐type Czochralski‐grown (Cz) silicon wafer is used to prepare MWCNTs: Nafion/Si solar cells. The device preparation is divided into several steps: 1) Wet chemistry was used to clean the back surface of the silicon wafer, while the front surface was composed of an Ag/SiN_x_/n^+^ emitter stack. 2) Spin‐coating at 3500 rpm of an MWCNTs: Nafion composite film on the backside under a N_2_ atmosphere at room temperature. 3) A 300 nm‐thick conformal Ag electrode was deposited on the MWCNTs: Nafion film by thermal evaporation.

### 4.3 Characterization

TEM images were performed on a Helios Nanolab G3 CX to characterize the surface morphology of the MWCNTs sponge. EDS mapping was conducted on a JOEL JEM‐F200 at an accelerating voltage of 200 kV to determine the interfacial element distribution. The size distribution of MWCNT particles in the ink was measured using the Malvern ZS90. The work function of the film was measured using the ESCALab250Xi type equipment (He I, 21.22 eV) of Thermo Fisher Scientific for ultraviolet photoelectron spectroscopy (UPS). The contact resistance was measured using the transmission line model (TLM). The conductivity was measured by the coplanar electrode method. The thickness of the MWCNTs: Nafion film was measured by a step profiler (Dektak XT) and SEM (Nova NanoSEM450, FEI). The minority carrier lifetime (*τ*_eff_) was measured using the transient photocurrent decay (PCD). Raman spectroscopy analysis was performed using the Horiba LabRam HR Evolution. The morphology of the silicon surface was characterized using SEM (Nova NanoSEM450, FEI). The solar cells were characterized by current density‐voltage (*J‐V*) measurements under standard conditions (AM1.5, 100 mW cm^−2^, and 25°C) and EQE (R3011, Enlitech). The light intensity was calibrated with a reference cell prepared by Fraunhofer ISE CalLab. The EQE was characterized by a SpeQuest QE 1226 (RERA) using monochromatic light from a Xenon arc‐discharge lamp.

## Author Contributions

**Jianhui Chen**: conceptualization, methodology, supervision, validation, resources, investigation, funding acquisition, writing – review and editing. **Anyuan Cao**: conceptualization, methodology, resources, validation, investigation, funding acquisition. **Qing Gao**: conceptualization, methodology, data curation, supervision, visualization, validation, investigation, funding acquisition, writing – original draft, writing – review and editing. **Xuan Chang**: data curation, methodology. **Tiansheng Sun**: data curation, validation. **Yiming Xu**: data curation, methodology. **Lu Zhang**: data curation, methodology. **Bingbing Chen**: funding acquisition. **Dehua Yang**: conceptualization, methodology, funding acquisition, resources. **Jianxin Guo**: methodology, software. **Jianan Zhang**: data curation, methodology. **Jiahe Chen**: data curation, investigation, validation, visualization. **Qi Wang**: writing – original draft, data curation, investigation, validation, resources, writing – review and editing, visualization. **Yuke Ren**: methodology, data curation, investigation, validation, writing – original draft, writing – review and editing, visualization.

## Conflicts of Interest

The authors declare no conflicts of interest.

## Supporting information




**Supporting File**: advs76174‐sup‐0001‐SuppMat.docx.

## Data Availability

Research data are not shared.
