# Supplementary material for: High‐Performance Multi‐Walled Carbon Nanotubes‐Organic Passivated Si Solar Cells Enabled by Spatially Selective Harvesting of High‐Quality Sponges
Source: Adv Sci (Weinh). 2026 Jul 10:e76174. Online ahead of print. doi: 10.1002/advs.76174 (PMC13353175; doi:10.1002/advs.76174)
Supplement: Supplementary file 1 — Supporting File: advs76174‐sup‐0001‐SuppMat.docx. [file ADVS-9999-e76174-s001.docx]

**High-Performance Multi-Walled Carbon Nanotubes-Organic Passivated Si Solar Cells Enabled by Spatially Selective Harvesting of High-Quality Sponges**

Yuke Ren^#^, Qi Wang^#^, Jiahe Chen, Jianan Zhang, Jianxin Guo, Dehua Yang, Bingbing Chen, Lu Zhang, Yiming Xu, Xuan Chang, Tiansheng Sun, Qing Gao*, Anyuan Cao*, and Jianhui Chen*

Y. Ren, J. Chen, J. Zhang, J. Guo, D. Yang, B. Chen, L. Zhang, Y. Xu, X. Chang, T. Sun, Q. Gao, J. Chen

Advanced Passivation Technology Lab College of Physics Science and Technology Hebei University Baoding 071002, China

E-mail: gaoqing@hbu.edu.cn; chenjianhui@hbu.edu.cn

Y. Ren, J. Chen, J. Zhang, J. Guo, D. Yang, B. Chen, L. Zhang, Y. Xu, X. Chang, T. Sun, Q. Gao, J. Chen

Province-Ministry Co-Construction Collaborative Innovation Center of Hebei Photovoltaic Technology College of Physics Science and Technology Hebei University Baoding 071002, China

E-mail: gaoqing@hbu.edu.cn; chenjianhui@hbu.edu.cn

J. Guo, D. Yang, B. Chen, Q. Gao, J. Chen

State Key Laboratory of Photovoltaic Materials and Cells, Yingli Group Co., Ltd, Baoding 071051, China

E-mail: gaoqing@hbu.edu.cn; chenjianhui@hbu.edu.cn

A. Cao

State Key Laboratory of Advanced Waterproof Materials, School of Materials Science and Engineering, Peking University, Beijing 100871, China

E-mail: anyuan@pku.edu.cn

Q. Wang

Henan Key Laboratory of Advanced Conductor Materials, Institute of Materials, Henan Academy of Sciences, Zhengzhou 450046, China

# These authors contributed equally to this work

**Supporting Data**

**Table S1.** Historical progression of MWCNTs:Nafion/Si solar cells efficiencies over the past few decades.

| Device Structure | *V*oc  (mV) | *J*sc  (mA cm^-2^) | FF  [%] | PCE  [%] | Year |
| --- | --- | --- | --- | --- | --- |
| MWCNT/n-Si | 290 | 2.7 | 46 | 3 | 2008^[1]^ |
| Ag/MWCNT-HNO_3_/n-Si | - | - | - | 1.39 | 2014^[2]^ |
| MWCNT-Graphene/n-Si | 560 | 30.88 | 52 | 9.24 | 2015^[3]^ |
| MWCNT/n-Si | 550 | 30.2 | 60 | 10 | 2016^[4]^ |
| MWCNT/n-Si | 498 | 33.17 | 44.96 | 7.42 | 2018^[5]^ |
| PEDOT: PSS /MWCNTs&PDA/n-Si | 637 | 34.76 | 56.41 | 12.49 | 2021^[6]^ |

**Table S2**. Statistical data (mean, standard deviation) of MWCNTs:Nafion/Si Solar Cells based on MWCNTs grown under different H_2_ flow rate and carbon source flow rate. Each condition contains 7 solar cells.

| **Mean ± Standard Deviation** | | | | |
| --- | --- | --- | --- | --- |
| **H_2_ flow rate - Carbon source flow rate**  **(mL/min — μL/min)** | ***V*_OC_ (mV)** | ***J*_SC_ (mA/cm^2^)** | **FF (%)** | **PCE (%)** |
| **80-110** | 656±1.53 | 38.51±0.71 | 82.87±0.70 | 20.95±0.33 |
| **180-110** | 663±2.45 | 39.29±0.28 | 83.28±0.43 | 21.69±0.20 |
| **380-110** | 669.6±4.24 | 39.79±0.12 | 83.98±0.66 | 22.38±0.22 |
| **480-110** | 665.6±5.83 | 39.65±0.26 | 84.16±0.73 | 22.28±0.34 |
| **380-200** | 669.6±5.68 | 39.86±0.34 | 84.13±0.43 | 22.44±0.31 |
| **380-290** | 664.3±3.35 | 39.15±0.44 | 84.27±0.53 | 21.77±0.33 |

**Supporting Figures**

**
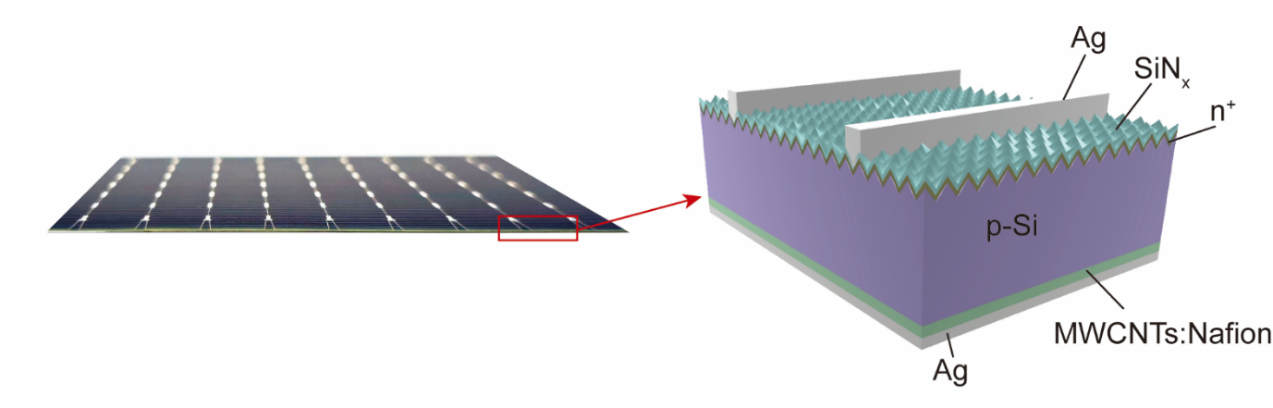
**

**Figure S1.** Schematic of the MWCNTs:Nafion/Si solar cells architecture used in this work.

**
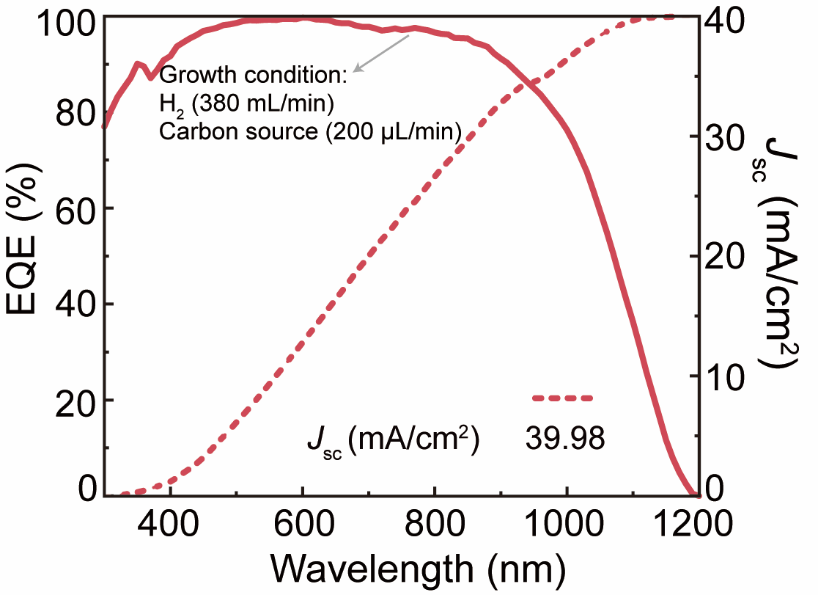
**

**Figure S2.** EQE curve of the MWCNTs:Nafion/Si solar cells under optimal photovoltaic performance.

**
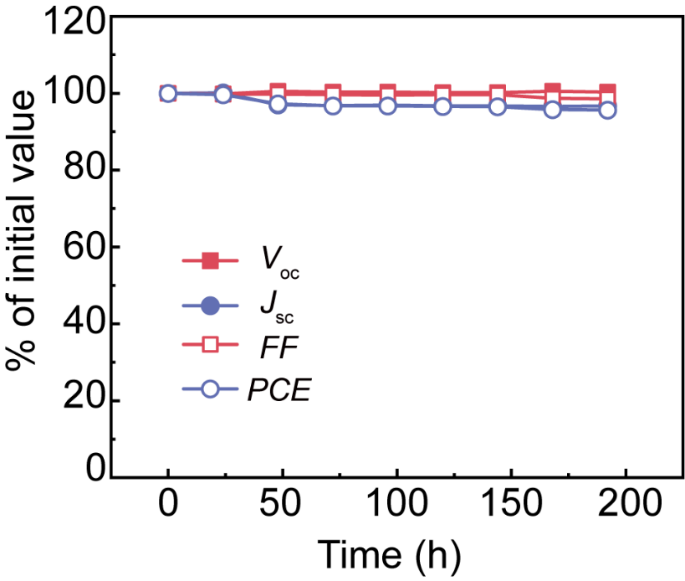
**

**Figure S3.** Device stability of MWCNTs:Nafion/Si solar cells.

**
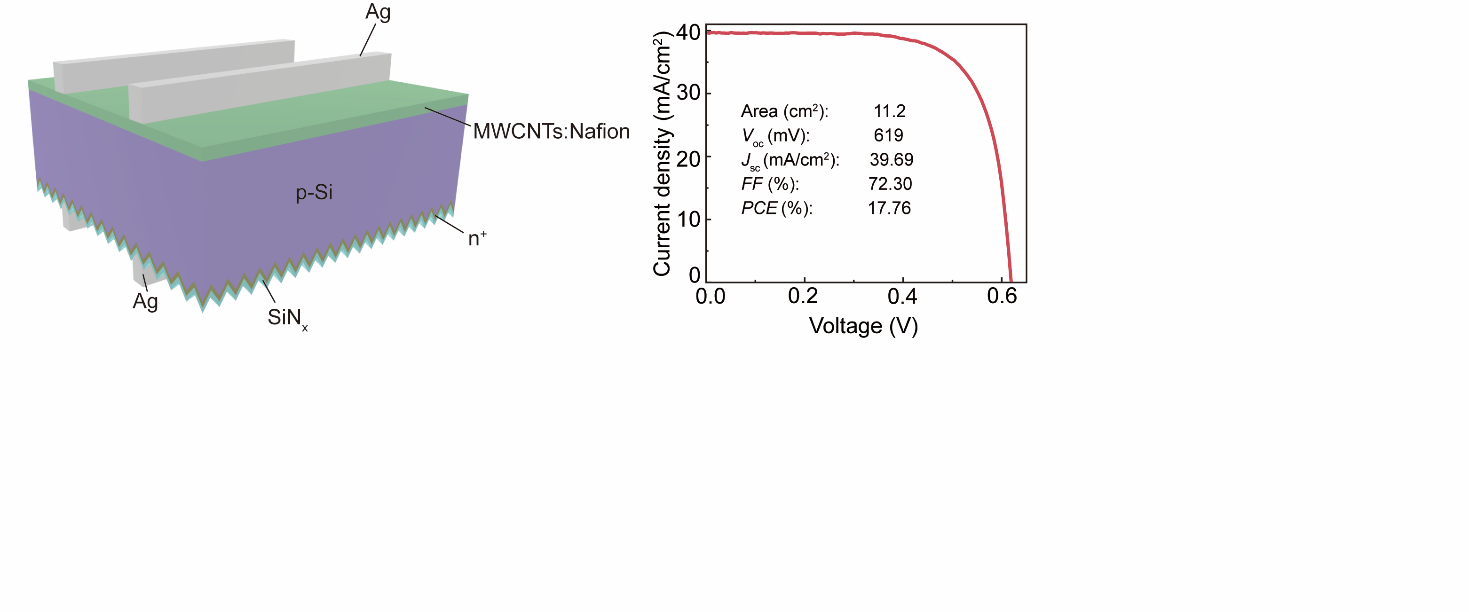
**

**Figure S4.** Schematic of device structure using MWCNTs:Nafion film as the transparent hole transport layer and the corresponding *J-V* curve and performance.

**
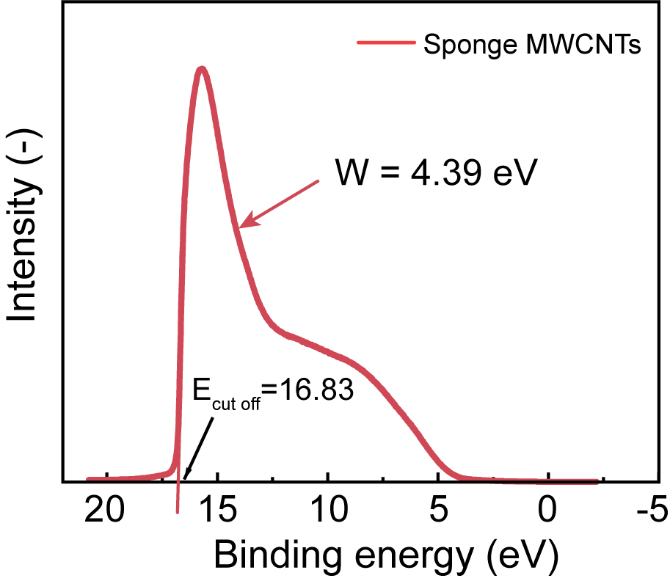
**

**Figure S5.** Work function of Sponge MWCNTs.


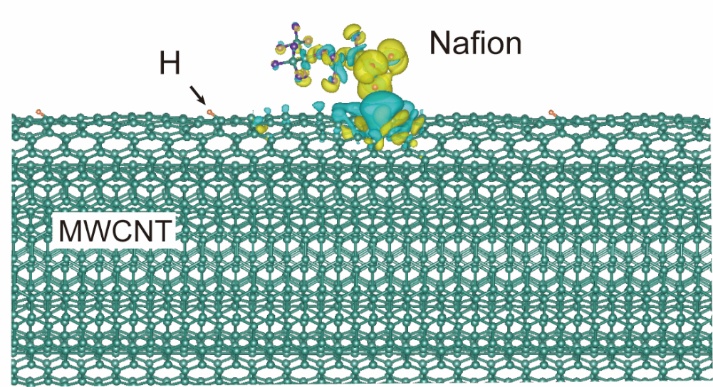


**Figure S6.** Calculated charge density difference for Nafion molecule binding with the MWCNT.

**
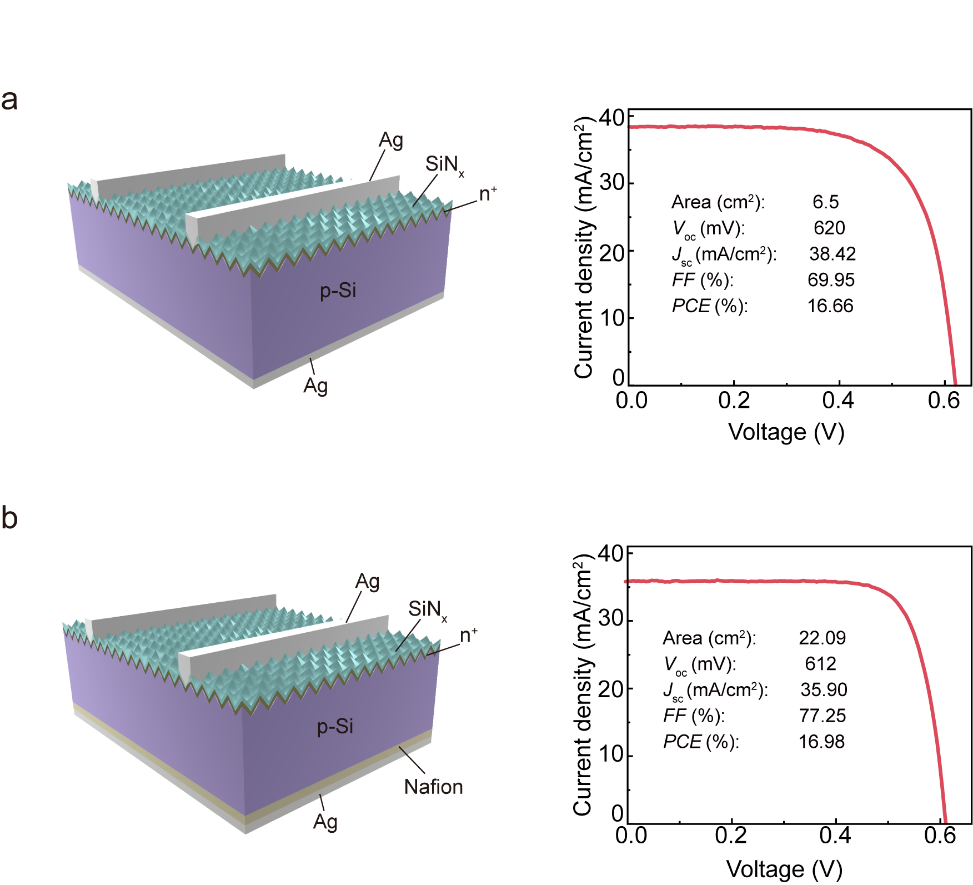
**

**Figure S7.** a) Schematic, *J-V* curve and performance of the reference solar cells without MWCNTs:Nafion film. b) Schematic, *J-V* curve and performance of the reference solar cells with a Nafion film replacing the MWCNTs:Nafion film.


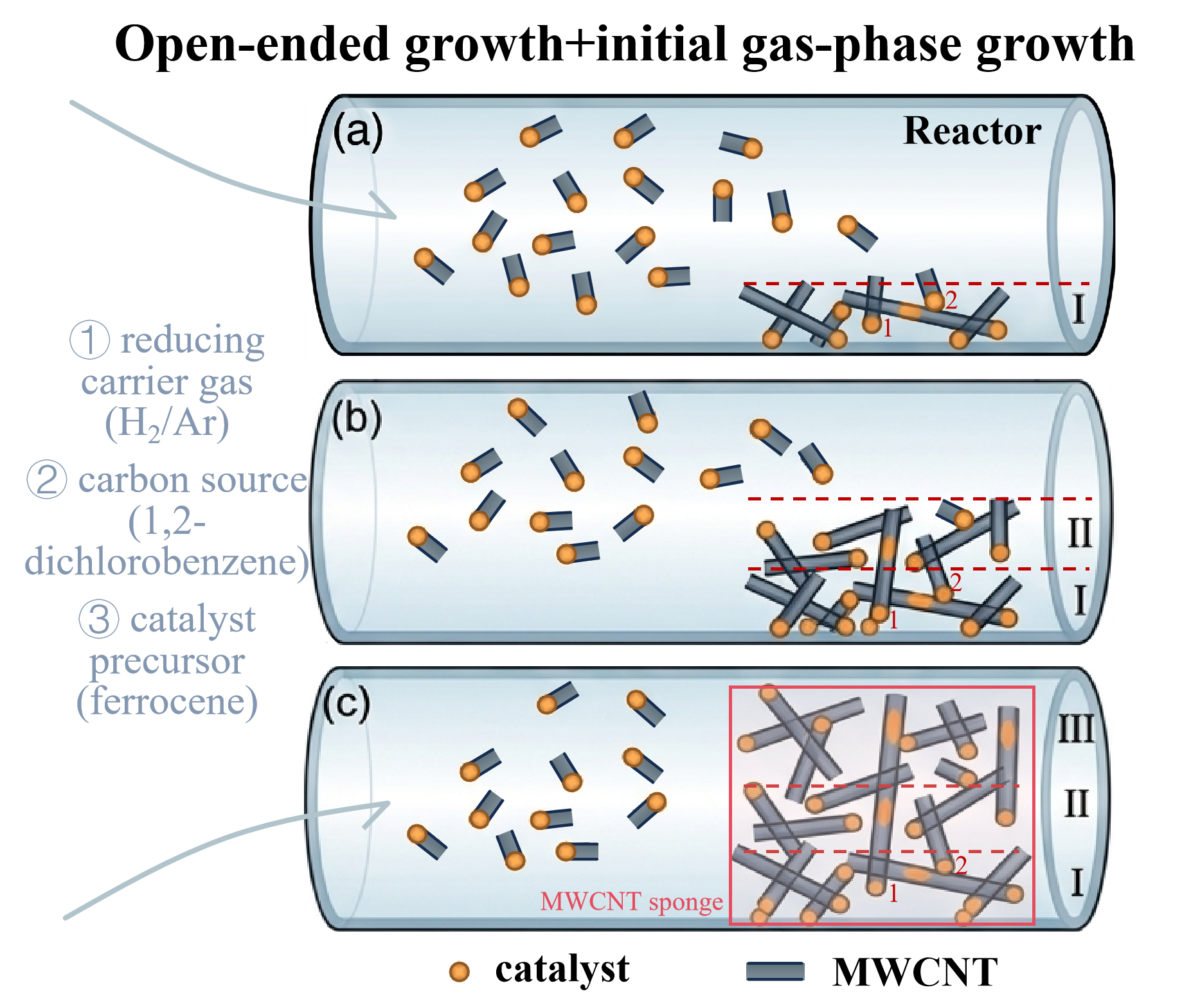


**Figure S8**. Schematic illustration of the growth mechanism of the MWCNT sponge.^[7]^


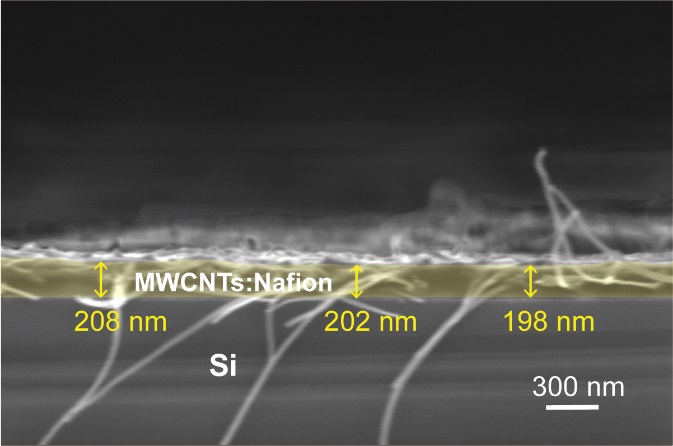


**Figure S9.** The cross-sectional SEM image the in-MWCNTs:Nafion film.

**Computational method:**

In this work, all density functional theory (DFT) computations were performed using the Vienna Ab initio Simulation Package (VASP) ^[8-9]^, based on the projector augmented wave (PAW) formalism^[10]^. To account for electron exchange and correlation, we adopted the generalized gradient approximation (GGA) within the Perdew–Burke–Ernzerhof (PBE) functional, along with van der Waals (vdW) corrections^[11]^. A uniform plane-wave cutoff energy of 400 eV was applied throughout. Given the large in-plane dimensions of the supercells (40 Å × 40 Å) along the a and b axes, a Monkhorst–Pack grid of 1×1×3 was considered adequate for Brillouin zone sampling. Geometry relaxations were carried out for multi-walled carbon nanotubes (MWCNTs) with a simplified Nafion molecular unit adsorbed onto their surfaces. The Bader charge analysis^[12]^ was conducted on the relaxed geometries to evaluate the charge transfer between the MWCNTs and the adsorbed Nafion units.

To characterize how a Nafion molecular unit adsorbs onto the MWCNTs surface, we define the adsorption energy (E_a_) as follows:

1. $E_{a}= E_{\mathrm{total}}- E_{\mathrm{MWCNTs}}-E_{\mathrm{Nafion}}$ (1)

In this equation, $E_{\mathrm{total}}$​ refers to the overall energy of the composite system comprising the MWCNTs and the adsorbed Nafion species, while $E_{\mathrm{MWCNTs}}$ is the energy of the bare MWCNTs, and $E_{\mathrm{Nafion}}$ stands for the energy of an isolated Nafion molecular unit (either in the presence or absence of ethanol, as depicted in Figure S6). According to this formulation, a negative $E_{a}$ value implies that the resulting adsorption structures are thermodynamically favorable.

**REFERENCES**

1. A. Arena, N. Donato, G. Saitta, S. Galvagno, C. Milone, A. Pistone, *Microelectron. J* 2008, 39 (12), 1659.
2. M. Mohammed, Z. Li, J. Cui, T.-p. Chen, *Sol. Energy* 2014, 106, 171.
3. S. Wu, E. Shi, Y. Yang, W. Xu, X. Li, A. Cao, *Nano Res* 2015, 8 (5), 1746.
4. F. De Nicola, M. Salvato, C. Cirillo, M. Crivellari, M. Boscardin, M. Scarselli, F. Nanni, I. Cacciotti, M. De Crescenzi, P. Castrucci, *Carbon* 2016, 101, 226.
5. S. Wu, L. Yang, H. Wu, Y. Yuan, Z. Ji, A. Cao, *ChemistrySelect* 2018, 3 (33), 973.
6. H. Guo, T. Chen, L. Yu, A. Chen, T. Sun, J. Wang, C. Wang, J. Zhang, Y. Yang, *Opt. Mater* 2021, 12.
7. X. Gui, J. Wei, K. Wang, A. Cao, H. Zhu, Y. Jia, Q. Shu, D. Wu, Adv. Mater. 2010, 22 (5), 617.
8. G. Kresse, J. Hafner, Phys. Rev. B 1993, 47 (1), 558,
9. G. Kresse, J. Furthmüller, Comput. Mater. Sci. 1996, 6 (1), 15.
10. P. E. Blöchl, Phys. Rev. B 1994, 50 (24), 17953.
11. S. Grimme, J. Antony, S. Ehrlich, H. Krieg, J. Chem. Phys. 2010, 132 (15).
12. G. Henkelman, A. Arnaldsson, H. Jónsson, Comput. Mater. Sci. 2006, 36 (3), 354.
